# Coronavirus Disease 2019 (COVID-19) Crisis Measures: Health Protective Properties?

**DOI:** 10.3390/medicines8090049

**Published:** 2021-09-02

**Authors:** Abdelaziz Ghanemi, Mayumi Yoshioka, Jonny St-Amand

**Affiliations:** 1 Functional Genomics Laboratory, Endocrinology and Nephrology Axis, CHU de Québec-Université Laval Research Center, Québec, QC G1V 4G2, Canada; abdelaziz.Ghanemi@crchudequebec.ulaval.ca (A.G.); mayumi.yoshioka@crchudequebec.ulaval.ca (M.Y.); 2 Department of Molecular Medicine, Faculty of Medicine, Laval University, Québec, QC G1V 0A6, Canada

**Keywords:** coronavirus disease 2019 (COVID-19), measures, positive, health

## Abstract

The ongoing 2019 coronavirus disease (COVID-19) crisis has led governments to impose measures including mask wearing, physical distancing, and increased hygiene and disinfection, combined with home confinement and economic shutdown. Such measures have heavy negative consequences both on public health and the economy. However, these same measures have positive outcomes as “side effects” that are worth mentioning since they contribute to the improvement of some aspects of the population health. For instance, mask wearing helps to reduce allergies as well as the transmission of other airborne disease-causing pathogens. Physical distancing and social contact limitation help limit the spread of communicable diseases, and economic shutdown can reduce pollution and the health problems related to it. Decision makers could get inspired by these positive “side effects” to tackle and prevent diseases like allergies, infectious diseases and noncommunicable diseases, and improve health care and pathology management. Indeed, the effectiveness of such measures in tackling certain health problems encourages inspiration from COVID-19 measures towards managing selected health problems. However, with the massive damage COVID-19-related measures have caused to countries’ economies and people’s lives, the question of how to balance the advantages and disadvantages of these measures in order to further optimize them needs to be debated among health care professionals and decision makers.

The current 2019 coronavirus disease (COVID-19) crisis represents a global health problem [1] with severe negative consequences on the economy as well as on public health (mental, physical, obesity, immunity, etc.) [2,3,4]. It is worth noting that these consequences are mainly due to the measures governments have imposed in order to limit the spread of the virus causing COVID-19. Among these measures, mask wearing and increased hygiene have almost only positive effects, while distancing, lockdowns and travel restrictions have serious negative effects. Indeed, the ongoing COVID-19 pandemic [1] has caused us to develop new lifestyle habits in terms of hygiene, health practices and social limitation. Within this context, media coverage and medical attention focus on following the negative health consequences of the COVID-19 pandemic, but not the “positive outcomes” resulting from this current health crisis. Such positive effects would represent “side effects”, they cannot outweigh the negative ones nor can they justify the measures if they are outside of a pandemic context. However, they remain worth mentioning to cover other aspects of the COVID-19 pandemic. Herein, we present illustrative examples of the benefits that the imposed measures can have on public health regarding diseases and health problems other than COVID-19. Such examples lead us to consider applying similar approaches (optimized) to tackle and manage various public health issues.

Hygiene habits including hand washing and/or hand sanitizing (alcohol sanitizer) [5,6] eliminate numerous pathogens and reduce health problems including gastroenteric and respiratory infections [5], diarrhea episodes [7], nosocomial pathogens [8] and healthcare-associated infections [9]. In addition, the increased cleaning and disinfection frequency both in houses and in public places eliminates many pathogens individuals usually encounter by touching surfaces and physical supports.

Confinement and lockdown [10], combined with physical distancing [11], reduce the interactions between individuals and limit the number of places (occasions) in which individuals gather. Therefore, the pathogens exchanged via human contact decreases. This is further strengthened by the limitation of travel both internationally as well as locally. In addition, many public places have been closed which also limits gathering possibilities at sites in which active transmission of pathogens can occur such as cinemas, theaters, museums, stadiums, and gymnasiums.

Besides infectious agents, the lockdowns and economic shutdowns, among other measures, may reduce the global pollution rate [12,13,14]. As is known, pollution is associated with diverse diseases [15] such as kidney diseases [16] and congenital anomalies [17]. Therefore, pollution reduction may have significant impacts on population health in terms of improving pollution-related health problems, especially in developing countries [18], and may prevent numerous diseases including various noncommunicable diseases [19,20]

Importantly, wearing masks (as a measure to prevent COVID-19 spread [21]), may also help to limit respiratory disease transmission such as flu, inhalation of some airborne pollutants, as well as exposure to allergy-inducing agents. Herein, it is important to highlight the increased awareness within the general population of how diseases can both be transmitted and prevented, which would be very significant considering the importance of population education in managing health problems [22].

As a conclusion, these observations represent properties worth exploring in an epidemiological context toward developing options to deal with health problems that may be improved with the measures taken during the ongoing COVID-19 crisis. In this context, we would also like to highlight the emergence of the wide-range use of computers and internet awareness, which was previously not common in rural areas of developing and underdeveloped nations and that has been pivotal throughout the pandemic. Now, with remote working it has become much easier to spread health related information in order to undertake the required preventions and precautions against the disease, increasing public awareness and health education even for professionals. This article does not aim to support the measures by ignoring the often-massive damage they have on countries’ economies and people’s lives. Rather, we aim to describe the positive effects as a “side effect” of the measures. Beyond these observations, the health benefits resulting from the measures taken by government and health officials to limit the spread of COVID-19 represent good models to focus on in promoting a healthy society (Figure 1).

Decision makers could mimic and get inspired by these positive “side effects” to tackle and prevent diseases like allergies, infections and noncommunicable diseases, and improve health care and pathology management. Indeed, the effectiveness of such measures in tackling certain health problems encourages inspiration from COVID-19 measures toward management of selected health problems. However, with the massive damages that COVID-19-related measures have done to countries’ economies and people’s lives, the question of how to balance the advantages and disadvantages of these measures needs to be debated among health care professionals and decision makers. The purpose of this work is to improve public health through implementing measures inspired by the handling of the COVID-19 crisis, while limiting the negative impacts we have seen.

## Figures and Tables

**Figure 1:**
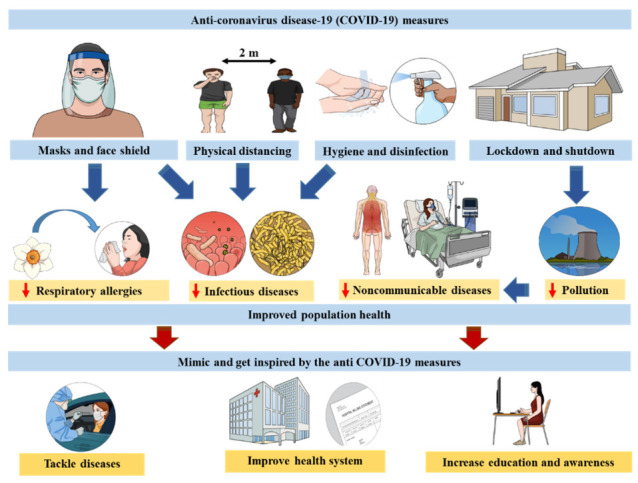
The positive outcomes of the measures taken to limit COVID-19 spread represent “side effects” of these measures. Yet, they are worth describing in order to use the related conclusions towards improving various aspects of health care.

## Data Availability

Not applicable.
